# Erratum

**Published:** 2022-11

In manuscript “Efficacy of interspace between the popliteal artery and the capsule of the posterior knee (iPACK) block versus periarticular local infiltration analgesia after unilateral total knee arthroplasty. Prospective randomized control trial” Saudi Med J 2021; Vol. 42 (10): 1065-1071 doi: 10.15537/smj.2021.42.10.20210504.

The affiliation should appeared as: From the Department of Anesthesiology (Narejo, Abdulwahab, Aqil, Alsubaie, Alzahrani, Thallaj); from the Department of Orthopedic Surgery (Aljurayyan), College of Medicine, King Saud University

The e-mail should appear as: ahaji@ksu.edu.sa

